# Melissopalynological analysis and microbiological safety of fresh and market honey (*Apis mellifera* L. and *Meliponulabeccarii* L.) from Western Oromia, Ethiopia

**DOI:** 10.1016/j.heliyon.2024.e28185

**Published:** 2024-03-19

**Authors:** Ofijan Tesfaye, Asnake Desalegn, Diriba Muleta

**Affiliations:** a Oromia Agricultural Research Institute, Haro Sebu Agricultural Research Center, Haro Sebu, Kellem Wollega, Oromia, Ethiopia; b Department of Microbial, Cellular, and Molecular Biology, Addis Ababa University, Collage of Natural and Computational Sciences, Addis Ababa, Ethiopia; c Institute of Biotechnology, Addis Ababa University, Collage of Natural and Computational Sciences, Addis Ababa, Ethiopia

**Keywords:** *Apis mellifera*, *Bees*, *Honey*, *Meliponulla beccarii*, Microbial qualities

## Abstract

Honey is a natural product that is made by bees from the nectar of flowering plants. There is a flora preference by bees. Like other foods ready to eat,honey can be prone to microbial contamination. Honey plant sources can be analyzed from the composition of pollen grains in honey samples. The objective of this study was to assess microbial safety and floral origin of the honey samples. For this study, honey samples were purchased from local market, and collected from hives (fresh honey) in Western Oromia. Floral analysis was determined using harmonized method of melissopalynology. Microbiological safety was assessed through the pour plate procedures from the first serial dilution on a total of 45 honey sample sizes.The melissopalynological analysis demonstrated that *A*. *mellifera*honey purchased from the market(AMMH) was considered a multi-floral type while *A*. *mellifera* fresh honey (AMFH) cropped directly from the hive and *M*.*beccarii* honey purchased from the market (MBMH) was dominated pollen from *Coffee arabica* (68 % of its pollen grain counted) and *Guizotiascabra* (50.53 % of its pollen grain counted) plant, respectively. The Aerobic mesophilic bacteria, Staphylococci, Yeast, Mould, and Aerobic spore-forming bacteria were found below the standard countable level (<30 cfu/plate) from *A*. *mellifera* and *M*.*beccarii* honey bought from the market, while *A*. *mellifera* honey collected directly from the hive became free of any microbial contamination. *C*.*arabica* and ***G*.*scabra*** are major honey plants and their honey can be harvested in February and **October,** respectively. Furthermore, *Vernoniaamygdalina*, *Eucalyptus* spp, *Combretummolle*, *Trifoliumruppelianum,* and *Syzgiumguineense* were honey plants analyzed from multifloral market honey even though, their pollen dominance varies. *M. beccarii* visits herbaceous flora whilst *A. mellifera* visits all floral types. The level of contamination of the honey samples from the study area was very low showing its safety.

## 1 Introduction

Honey is naturally made by honeybees (*A. mellifera*) and stingless bees (*Meliponula* and *Trigona* spp.) fromthe nectar of flowers [1]. In Africa, *Trigona* and *Meliponula* are found under the genera of Meliponiae whilst in America they are under the genera of *Melipona* [2]. They are wild and have no sting unlike domesticated honeybees in the hive [3]. In Ethiopia, *Trigona* species and *M.beccarii* make their honey in tree trunks and below the underground, respectively [4], and their honey has more medicinal value [5].

Each species of plant has a unique structural pattern and genetic patternthat allow it to be distinguished from other species by its pollen grains [6,7]. For this, melissopalynological analysis has been used to examine the likely floral source of honey and it's more accurate than a visual survey to identify honeybee forages.Additionally, it offers details on the sources of nectar and pollen that honeybees in the area use to produce honey [8]. Honey might have multiple flowers (Multifloral honey) or just one (Monofloral honey). While multi-floral honey incorporates pollen from several plant species, monofloral honey is mostly generated from the pollen of a single plant species [7,9].

As with other ready-to-eat food, honey could be contaminated by microbes during pre and post-harvest handling. These microbes are those that can survive the intrinsic composition of honey. The honey microbial safety which is harvested from the hive by experienced beekeepers may be disparate from the markets. Ordo et al. [10], have demonstrated that post-harvest handling; transportation, adulteration, storage condition, materials used, and duration of storage could alter its nutritional quality and facilitate microbial growth [10].Common microbes that survive in honey are yeast and spore former bacteria [11, and microorganisms like fecal and total coliforms can develop in honey and cause human illness.

Even though the intrinsic environment of honey is not conducive tothe multiplication and survival of bacteria, few pathogens have been detected in honey [12]. *Clostridium botulinum* was identified in honey and is accountable for the growth of botulism in children or people with weakened immune systems [13]. *Bacillus cereus* is another pathogen that produces enterotoxin at pH 6.0–8.0 and temperatures ranging from 6 °C to 21 °C [14]. In Ethiopia there is no standard regulation/legislation on the microbial loads in honey sample. However, in Mexico(NMX-036-NORMEX-2006)a non mandatory standard was elaborated and stated that the presence of no more than 1000 CFU/g of non pathogenic bacteria and up to 100 CFU/g of yeast and mould shouldbe accepted [15]. In terms of biological loads, honey samples from the market may hold much more microbial loads than fresh honey in the hive. Honey at the market level may be contaminated with foreign material and have the characteristics of absorbing substances from its environment especially when the container is not closed well or harvesting uncapped honeycomb changes the quality and more water content brings to fermentation. Hence this work was proposed to identify the floral source and compare the microbial safety of honey samples at the market level and fresh honey harvested directly by bee technicians from the hive, and it was presented at Addis Ababa University, Ethiopia [16].

## 2 Materials and methods

### 2.1 Sample assortment

**Fig. 1:**
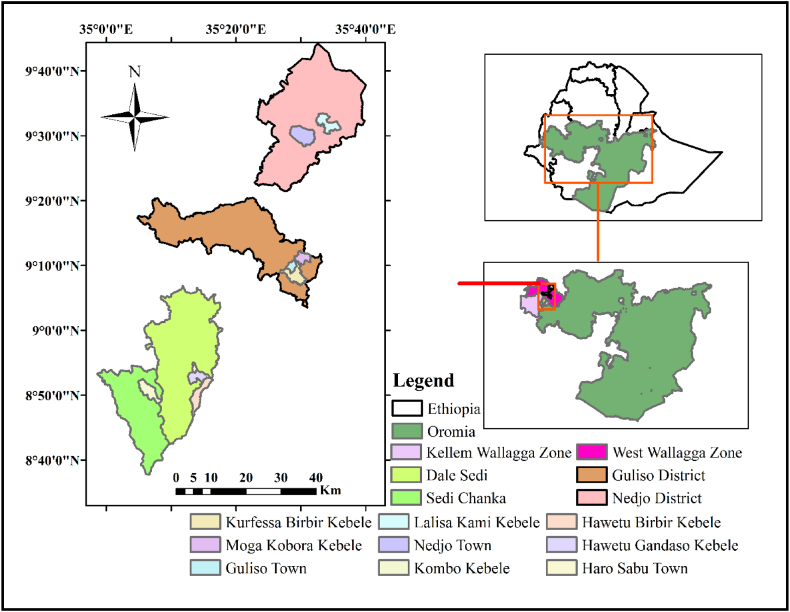
Map with geographical location of the study area.

The study was conducted in the West and Kellam Wollega Zones of Western Oromia, Ethiopia. Then, Dale Sedi Woreda (Hawetu gandaso and Hawetu birbir kebeles), and Sedi Chanka Woreda (Kombo kebele) from Kellem Wollega Zones whereas, Nedjo Woreda (Lalisa qami kebele) and Gulisso Woreda (Moga kobore and Kurfessa birbir kebeles) from West Wollega Zone were selected based on the potential and availability of honey produced by different entomological source. Map showing the honey sample collection areais depicted in Fig. 1. The geographic position of the sample collection area was depicted in Table 1. Three different groups of honey samples were used. The first group was honey from *A. mellifera*, bought from the honey market/shop of Haro Sebu and Gulisso town and assigned as *A. mellifera* market honey sample (AMMH). Harvesting time, post harvest processing and handling, botanical and geographical origin of honey which was purchased from honey shop is unknown since it's a mixture from different places by retailers/whole sellers/local collectors. The second group was *A. mellifera* honey directly harvested from modern hive (moveable frame hive) at apiary site of Hawetu Gandaso, Kombo, Moga kobore and Kurfessa birbir and assigned as *A. mellifera* fresh honey sample (AMFH). Capped/sealed honeycombs were selected and harvested by bee technicians using sterile materials considering a contamination. The third group was honey from *M. beccarii* (underground honey) purchased from Nedjo town and farmers of Lalisa kami kebele which has high potential area for *M. beccarii* honey production and assigned as *M. beccarii* market honey sample (MBMH). Then, for each group, 15 kg separately; kg/sample, a total of 45 kg of both groups of samples were collected randomly. The collected honey samples were transported to the Lab. workplace, using cleaned and sterilized glass containers and their microbial load was analyzed in 2020.

**Table 1:** Description of the study area for topographical positions

Parameters	The study area (in kebele or peasant association)					
	Hawetu gandaso	Hawetu birbir	Kombo	Lalisa qami	Moga Kobore	Kurfesa birbir
Latitude	08°53′52''N	09°54′53''N	09°57′54''N	09° 38′41''N	09° 39′ 42''N	09° 31′41″ N
Longitude	35°14′19''E	35°15′19''E	35°16′20''E	34° 42′41''E	34° 43′42''E	34° 43′42''E
Altitude (m.a.s.l.)	1528	1575	1665	2062	1810	1726

### 2.2 Melissopalynological analysis

Pollen analysis of honey was carried out using harmonized method of melissopalynology [9,17]. For this, 10 g of honey was dissolved in 20 ml of warm distilled water in a centrifuge tube at temperatures that ranged from 20 to 40 °C. The solution was centrifuged at 1000 g for 10 min and the supernatant was decanted. The distilled water of 20 ml again was added to completely dissolve the remaining sugar crystals and centrifuged at 1000 g for 5 min and the supernatant was removed completely. The sediment was spread evenly using a sterile micro spatula on a microscope slide and the sample was dried for a while. Thereafter, one drop of glycerin jelly was added to the cover slip, and the pollen grains morphology from honey was identified using a pollen atlas prepared from the local flora of Ethiopia [18]. In addition to the pollen analysis, sensory properties, harvest time, and geographic regions were considered to differentiate the honey types. In addition to face-to-face communication with highly experienced beekeepers in each study area, visual observation of the apiary environment was conducted during harvest to identify the flower source of honey samples and bee forages growing in the area. The percentage of pollen types in each honey sample was calculated based on the total number of different types of pollen grains counted in each sample. Then pollen was categorized as predominant pollen (monofloral honey), if the relative frequency of the pollen of that *taxon* exceeds 45%, secondary pollen (16–45%), important minor pollen (3–15%), and minor pollen (<3%) according to standard reference by Louveaux *et al.* [9]. The pollen count was done under a light microscope (Swift instrument international, serial number 8750038, Japan, high power 400x) linked to a computer. Pollen grain analysis of the honey samples was done at the Laboratory of Holota Apiculture Research Center and replicated three times.

### 2.3 Microbiological quality of honey samples

Microbial counts of aerobic mesophilic bacteria, staphylococci, Enterobacteriaceae, Coliforms, aerobic bacterial spore formers, yeast, and mould were analyzed using standard method [19]. For this purpose, 20 g of sample was addedto 180 mL of sterilized distilled water and shakenviaan orbital shaker. A 0.1 ml from 10^−1^ serial dilution was poured onto the center of the sterilized respective plate media. 20–25 ml of the sterilized respective media was tipped onto the plates comprising the aliquots and well mixed. The inoculated plates were incubated at appropriate temperature for 24–96 h. The colonies were enumerated from countable plates (a plate containing between 30 and 300 CFU/plate) and expressed as colony forming units per gram or ml. Accordingly; the following group of microbes were counted.

#### 2.3.1 Aerobic mesophilic counts

From 10^−1^ serial dilution, a 0.1 ml of aliquot was poured in duplicate onto plates of Nutrient Agar (Oxoid) and the colonies were counted after the plates were incubated under aerobic condition at 30–32 °C for 24–48 h [19].

#### 2.3.2 Staphylococci counts

From appropriate serial dilution, 0.1 ml of aliquot was poured in duplicate onto plates of Mannitol Salt Agar (Oxoid). The plates were incubated under aerobic condition at 30–32 °C for 36 h. After incubation, yellow colonies with yellow zones and colorless or red colonies with red zones was counted as staphylococci [19].

#### 2.3.3 Enterobacteriaceae counts

From 10^−1^ serial dilution, 0.1 ml of aliquot was poured onto plates of violet red bile glucose agar (Oxoid) (g/l: peptone-source of nitrogen, 7.00 g; yeast extract, 3.00 g; agar, 12.00 g; glucose, 10.0g; sodium chloride, 5.00 g; bile salts, 1.50 g; neutral red-as dye, 0.03 g; Crystal Violet, 0.002 g; Distilled water, 1000 ml with final pH of 7.4 ± 0.2. Colonies were counted after the plates incubated under aerobic condition at 30–37 °C for 18–24 h and after which, pink to red purple colonies were considered as member of the family Enterobacteriaceae [19].

#### 2.3.4 Coliforms

From 10^−1^ serial dilution, 0.1 ml of aliquot was poured onto plates of violet red bile agar (Oxoid) (lactose, 10.0 g; peptic digest of animal tissue, 7.0 g; sodium chloride, 5.0 g; yeast extract, 3.0 g; bile salts mixture, 1.5 g; neutral red, 0.03 g, crystal violet, 0.002 g; agar, 15.0 g; distilled water, 1000 ml with pH of 7.4 ± 0.2. Colonies were counted after the plates incubated under aerobic condition at 30–32 °C for 20–24 h [19].

#### 2.3.5 Yeasts and molds

From 10^−1^serial dilution, 0.1 ml of aliquot was poured onto plates of Potato Dextrose Agar (Oxoid) containing (g/l) potatoes infusion, 200.00 g; dextrose, 20.00 g; agar, 15.00 g; chloramphenicol, 0.1 g; distilled water, 1000 ml with pH value of 5.6 ± 0.2 and incubated at 2528 °C for three to five days. Smooth (non-hairy) colonies without extension and hairy colonies with extension at periphery were counted as yeasts and molds, respectively (19).

#### 2.3.6 Aerobic bacterial spore formers

A 10^−1^ ml of the serial dilutions was heated in a water bath at 80 °C for 10 min to kill vegetative cells and cooled rapidly in tap water. A 0.1 ml of aliquot was poured onto plates of Nutrient Agar (Oxoid). The grown colonies were counted as aerobic spore former bacteria after incubation at 30–32 °C for 48 h (19).

The number of bacterial and fungal growth were then counted using a colony counter and the results were recorded and expressed as colony-forming units per gram of the sample using theequation 1 indicated belowsince the colony load was less than 30 per plate, it unnecessary to identifythe microorganisms [20]. Microbial quality analysis were carried out at Addis Ababa University Department of Microbial, Cellular and Molecular Biology (Applied Microbiology), and Food Science and Nutrition Laboratories and replicated three times.

1 $$\frac{cfu}{g}=\frac{Total\;number\;of\;colonies\;counted\;X\;dilution\;factor\;}{Volume\;of\;inocula\;}$$

Where:

cfu = colony forming unit

g = gram.

### 2.4 Data analysis

All the data were analyzed by using Microsoft Excel and presented in the table.

## 3 Results and discussion

### 3.1 Melissopalynological analysis (floral origin)

**Fig. 2:**
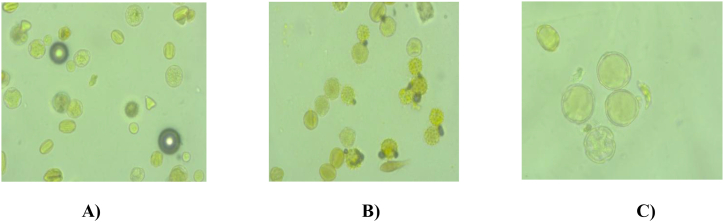
Pollen grain morphology identified from honey samples.A) *A*. *mellifera* market honey sample (Multifloral honey pollen), B) *A. mellifera* fresh honey sample (*C.arabica;* Monofloral honey),and C) *M*.*beccarii* market honey sample (*G.scabra)*.

The relative frequency of enriching nectariferous plant species and pictures of pollen grain morphology are depicted in Table 2 and Fig. 2 respectively. AMMH sample was considered as multi-floral (mixed) honey since each plant species found in honey samples wasbelow 45% pollen count. The plant species found in multi-floral honey were *G*.*scabra*, *C*.*arabica*, *V*.*amygdalina*, *Eucalyptus* spp, *C*. *molle*, *T*.*ruppelianum,* and *S*.*guineense*. This might be because the honey found at the commercial level is a mixture of the different botanical and geographical origins of honey or it is blended with honey harvested from different seasons by honey traders. On the other hand, the AMFH sample was dominated by *C*.*arabica* which could be found as monofloral honey since its pollen count was 68 %. *C*.*arabica* is a common cash crop in western Oromia, particularly in the study area, and contributed much to the monofloral nature of the harvested honey samples in February. This is supported by field observation and personal communication with local experienced beekeepers who stated that *C*.*arabica* flowers provide abundant pollen and nectar in January for honeybees [21]. The dominant pollen grains counted in MBH samples were *G*.*scabra* and *P*.*lanceolate* accounting for 50.3 and 44.5%, respectively. Honey from stingless bees (*M*.*beccarii*) is harvested once annually in October unlike honeybee honeys (*A*. *mellifera*). From the pollen analysis, *M*.*beccarii* visits herbaceous plants compared to *A*. *mellifera* which forages all herbs, shrubs, and tree plants. Alvareez-suarez *et al* [22] have demonstrated the presence of a difference between stingless and *A*. *mellifera* on foraging flowers due to the short flight rate of stingless bees or the difficulty of access to flowers used by *A*. *mellifera*. Most of the stingless bees nest on cultivated fields and fallow land that favor the growth of herbaceous flora. On the other hand, stingless bees are unable to compete with *A*. *mellifera* [23]. Moreover,the specialty of stingless bees is the ability to pollinate small-sized flowers due to their diminutive figure which cannot be achieved by the relatively big honeybee, and besidesstingless bees are not selective in building a colony hive [24].

**Table 2:** Relative frequency of enriching nectariferous plant species among the tested honey samples (% distribution).

Honey sample	Genus/Species name	Family name	Vernacular name (Afan Oromo)	Life form	Pollen grain counted (%)	Frequency class	Honey type
*A*. *mellifera* honey purchased from market; *A*. *mellifera* market honey (AMMH)	*G*.*scabra*	Asteraceae	Tuufoo	Herb	13.7	Significant minor pollen	Multifloral honey
	*C*.*arabica*	Rubiaceae	Buna	Shrub	11.3	Significant minor pollen	
	*V*.*amygdalina*	Asteraceae	Eebicha	Shrub	17.2	Secondary pollen	
	*Eucalyptus* spp.	Myrtaceae	Baargamoo	Tree	21.5	Secondary pollen	
	*C*.*molle*		Dhandhansa	Tree	12.9	Significant minor pollen	
	*T.ruppelianum*	Fabaceae	Siddisa	Herb	8.6	Significant minor pollen	
	*S*.*guineense*	Myrtaceae	Baddeessaa	Tree	14.6	Significant minor pollen	
*A*. *mellifera* honey harvested from hive; *A*. *mellifera* fresh honey (AMFH)	*C.arabica*	Rubiaceae	Buna	Shrub	67.9	Predominant pollen	Monofloral honey from *C. arabica* plant
	*G*.*scabra*	Asteraceae	Tuufoo	Herb	16.5	Secondary pollen	
	*Eucalyptus* spp.	Myrtaceae	Baargamoo	Tree	5.8	Significant minor pollen	
	*T*.*ruppelianum*	Fabaceae	Siddisa	Herb	5.8	Significant minor pollen	
	*V*.*amygdalina*	Asteraceae	Eebicha	Shrub	2.9	Minor pollen	
*M*.*beccarii* honey purchased from market (MBMH)	*G*.*scabra*	Asteraceae	Tuufoo	Herb	50.3	Predominant pollen	Monofloral honey from *G. scabra* plant
	*P. lanceolate*	Rubiaceae	Qorxobbii	Herb	44.5	Secondarypollen	
	*T*.*ruppelianum*	Fabaceae	Siddisa	Herb	5.2	Significant minor pollen

### 3.2 Microbiological profiles of honey samples

The microbiological safety of different groups of honey samples is depicted in Table 3. From *A. mellifera* market samples the counted microbes ranged from 2.1 × 10^3^ cfu/g by *Staphylococci* spp. to 1.17 × 10^2^ cfu/g by spore-forming bacteria. More yeast (3.16 × 10^2^ cfu/g) and fewer molds were counted from *M. beccarii* honey at the market level. However, Enterobacteriaceae and Coliforms were not observed from the examined samples which indicates the nonexistence of fecal adulteration in the samples and the sanitary status of honey on sale or there might be fecal contamination prior, but the organisms couldn't survive owing to incompatibility or the inherent features of the honey samples. Ethiopia has no honey quality regulation/legislation of microbial loads and moreover,as a worldwide this information is very limited on the literature. However, honey can have an acceptable quality with a bacterial count of up to 10000 CFU/g of aerobic mesophilic bacteria if they are free of fecal contamination [25] and this is comparable with the current result. Furthermore, a non-mandatory standard was elaborated in Mexico (NMX-036-NORMEX-2006) and stated that the presence of no more than 1000 CFU/g of non-pathogenic bacteria and up to 100 CFU/g of yeast and mould should be accepted [15]. The acidic condition and low water activity ofthe honey sample were responsible for the reduced survival of microorganisms. Honey samples which were collected directly from the hive were free from contamination. In comparison with *A.mellifera* honey from the market, the microbial load recorded from *M. beccarii* honey from the market was low. This might be due to the more acidic nature of honey made by *M. beccarii*, which as low pH and high free acidity that affects microbial growth in honey [12].

**Table 3:** Microbial loads among groups of honey.

Microbial load	Loads (colony forming unit per gram of honey)		
	*A. mellifera* market honey	*A. mellifera* fresh honey	*M. beccarii* market honey
Aerobic mesophilic bacteria	2.2 x 10^2^	NO	2.6 x 10^2^
Staphylococci bacteria	2.2 x 10^3^	NO	1.6 x 10^2^
Yeasts	2.0 x 10^3^	NO	3.2 x 10^2^
Molds	3.1 x 10^2^	NO	1.1 x 10^2^
Spore forming bacteria	1.2 x 10^2^	NO	1.6 x 10^2^
*Enterobacteriaceae* family	NO	NO	NO
Coliforms bacteria	NO	NO	NO

NO: Not observed (no microbes detected).

The microbial load from the market honey samples (*A. mellifera and M. beccarii*) of the current finding was less than the load from commercial honey samples reported in Ethiopia [11]. The study by Natea et al. [11] reported mean of 2.3 × 10^4^ cfu/g (aerobic mesophilic), 4.2 × 10^3^ cfu/g *(Staphylococci*), 7.4 × 10^3^ cfu/g (aerobic bacterial spore formers), 4.6 × 10^2^ cfu/g (yeast), 6.0 × 10^2^ cfu/g (molds) but no coliforms. Similarly, in the Nigeria market [19], the counting of bacteria ranged from 1.0 × 10^4^–2.0 × 10^4^ cfu/ml, yeast 1.0 × 10^4^–1.2 × 10^5^ cfu/ml, and 2.0 × 10^4^ cfu/ml coliform were observed from a sample. Moreover, from marketable honey of Portugal; Gomes et al. [26] have documented an average of <20 cfu/g of aerobic mesophilic bacteria, <22 cfu/g of fungi, and no fecal coliforms. Our study is comparable with marketable honey from Nigeria [19] which stated the absence of microbes from freshly collected honey related to some level of contamination for samples collected from the market place and other retail outlets. Like our finding, in honey from Romania [27], microbes were not detected in the samples harvested directly from the modern (moveable frame) hive, while *Bacillus* species and eight types of fungi were identified from honey samples from local markets. However, none of the above literature indicates whether the result fits with the microbial quality standard of the country and there is a shortage of information on the journal that mentions the normative microbial quality standard of honey worldwide or at a country level. The current finding points out that the hygienic way during a hive operation/inspection, honey extraction during harvest, and handling in the apiaries were rather capable, while at the market level honey contamination originates during post-harvest handling such as at processing, packing, storage, or planned contamination with foreign material. Fresh honey samples directly harvested by bee technicians of our study demonstrated microbial safety of the honey samples. This might be because, during harvesting, the capped honeycomb was selected by bee experts with sterile material for extraction and storage. Well-kept honey affords unfavorable environments for microbes to persist and replicate [28]. The existence of a small number of microbes in the samples is reflected to be related to contamination during straining, transport, packing, storing, or selling [29]. Comparatively as related to other microorganisms, a larger population of *Staphylococci* was obtained from market place samples which could be an indicator of poor post-harvest handling and contact with bare hands. *Staphylococci* are usually part of the skin flora and can be spread from individual to product by unsanitary practices [30]. Moreover, Al-waili et al. [31], verified the presence of high figures of vegetative bacteria owing to recent contamination but cannot reproduce in it, due to the honeys bacteriostatic effect.

### 3.3 Limitations of the study

The current study focused only on identifying the sources of honey plants from honey samples and assessed microbial loads between honey samples directly harvested from hives and purchased from honey shops limited to western Oromia, Ethiopia. However, it didn't examine the biological activities of the sample by collecting honey samples across the country owing to budget limitations.Therefore, future studies on the microbial safety of honey based on storage material and time, harvesting season, and hive types are necessary. Besides, the identification and documentation of honeybee floral sources regarding entomological and geographical origin across the country is vital.

## 4 Conclusion

From the present study, *C.arabica* is the major honey plant, and its honey is harvested in February, while *G.scabra* and *P.lanceolate* are the two most attractive forages and their honey is harvested in October. Furthermore, *G.scabra*, *C.arabica*, *V. amygdalina*, *Eucalyptus* spp, *C.molle*, *T.ruppelianum,* and *S.guineense* were honey plants analyzed from multifloral market honey even though, their pollen dominance varies.

Microorganisms were detected from samples purchased from the local market (*A.mellifera* and *M.beccarii* honey) even though the counts were below <30 cfu/ml. Microbes were not detected from the *A.mellifera* honey freshly collected directly from the hives. However, the presence of a certain number of bacteria from market honey samples is associated with contamination during straining, transportation, packaging, storage, or marketing.

## Funding

No special funding was provided for this project.

## Ethics approval and consent to participate

Not applicable.

## Availability of data and materials

The datasets used and/or analyzed during the current study are available upon reasonable request from the relevant author.

## CRediT authorship contribution statement

**Ofijan Tesfaye:** Writing – review & editing, Writing – original draft, Investigation, Data curation, Conceptualization. **Asnake Desalegn:** Writing – original draft, Visualization, Supervision, Project administration, Data curation. **Diriba Muleta:** Writing – original draft, Visualization, Supervision, Project administration, Conceptualization.

## Declaration of competing interest

The authors declare that they have no known competing financial interests or personal relationships that could have appeared to influence the work reported in this paper.
